# Genome-wide DNA methylation analysis of *Medicago sativa* L. treated with plasma and plasma-activated water

**DOI:** 10.1016/j.isci.2025.111901

**Published:** 2025-01-25

**Authors:** Fei Xu, Hao Chen, Chan Chen, Jiaqi Liu, Zhiqing Song, Changjiang Ding

**Affiliations:** 1 College of Science, Inner Mongolia University of Technology, Hohhot 010051, China; 2 Discharge Plasma and Functional Materials Application Laboratory, Hohhot 010051, China

**Keywords:** Plant biology, Interaction of plants with organisms, Plant Genetics

## Abstract

To explore the effects of plasma and plasma-activated water on whole-genome DNA methylation, in this study we used the *Medicago sativa* L. cultivar, as the experimental material, and mutant plants were obtained after treatment and screening. Changes in whole-genome DNA methylation in *Medicago sativa* L. were analyzed before and after mutagenesis using whole-genome bisulfite sequencing (WGBS) technology. We found that the percentage of methylated cytosines varied depending on the local sequence context (CG [dinucleotide context]), CHG and CHH (non-CG contexts, H is A [adenine] or T [thymine] or C [cytosine]) and external treatment. Differential methylated region (DMR) analysis revealed 41067 (CG), 5379 (CHG), and 257 (CHH) differentially methylated genes. This study quantitatively measured methylation levels, methylation sites, differentially methylated regions (DMRs), distribution of methylation in the genome, and methylation-related genes and pathways, for further investigations of the mechanism of plasma-induced mutagenesis.

## Highlights

• After mutation treatment, the survival rate of seed can be reduced to as low as 9%.
• There were also significant changes at the molecular level before and after treatment.
• After treatment, the roots of the seedlings grow rapidly.

## Graphical abstract

**Figure:**
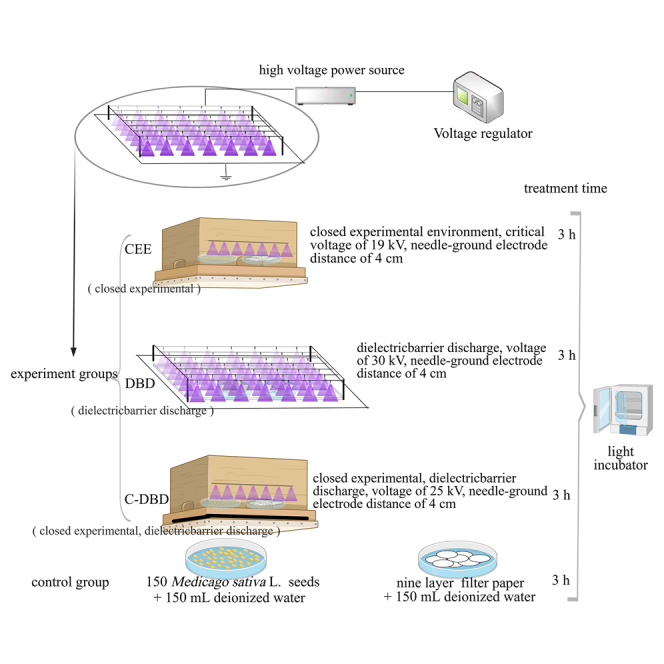

## Introduction

*Medicago sativa* L. is a kind of forage that is widely cultivated worldwide and is known as “the King of Forages”.^1^ It has the characteristics of high yield, high protein content, high-quality dietary fiber, balanced nutrition, strong biological nitrogen fixation ability, and strong adaptability. Therefore, it plays an irreplaceable role in promoting the sustainable development of animal husbandry and grass farming. It is an excellent grass species generally recognized worldwide, and it is also the most widely distributed herbaceous plant.^1^ However, at present, the degradation of natural grasslands leads to a decrease in forage yield and quality, and the imbalance between grass and livestock is becoming increasingly serious, which seriously limits the sustainable development of animal husbandry. Therefore, the genetic breeding of *Medicago sativa* L. is highly important.

In recent years, plasma has been widely used to improve seed vitality,^2^ fruit and vegetable freshness,^3^ disease control after fruit harvesting,^4^ plant protein modification,^5^ etc. However, there are few reports on molecular changes caused by artificial mutagenesis and the selection of new varieties using plasma. In addition to changes in DNA sequence, previous findings have shown that plant responses to stress can be attributed to changes in the state of chromatin, the structure of which can be rapidly and reversibly modified by insertion of methyl groups. Such modifications that alter gene expression without interfering with nucleotide sequences have been termed epigenetic changes.^6^ Epigenetics can affect plant growth and development by regulating gene expression, and DNA methylation is a type of epigenetic research that plays a crucial role in regulating plant growth and development, plant response to biotic or abiotic stress, and maintaining genome stability.^7^

DNA methylation is capable of altering gene expression without changing the DNA sequence and is one of the most important modes of epigenetic regulation. It is one of the most important epigenetic regulation methods. Plant DNA methylation plays an important role in regulating gene expression and cell differentiation.^8^ Studies have shown that in response to abiotic stresses such as high temperature, low temperature, drought, salinity, and heavy metals, plants regulate the expression of stress resistance genes through changes in DNA methylation levels of genome-wide or specific genes to better adapt to the survival environment.^9^ For example, RNA-mediated DNA methylation pathway-mediated DNA methylation dynamically regulates the expression of a number of heat stress-responsive genes^10^; cytosine methylation occurs in apples and is associated with water deficit^11^; and salt-induced expression of the AtMYB74 transcription factor is silenced by RdDM under normal conditions but activated after salt treatment.^12^ However, most of these studies have been aimed at understanding DNA methylation occurring under abiotic stresses such as temperature and soil salinization, and no studies have been conducted to assess the effects of plasma and its plasma-activated water on *Medicago sativ*a L. methylation. This study provides a scientific basis for the molecular mechanisms of epigenetic changes under mutagenic conditions.

Sequencing and array-based methods allow for studying DNA methylation across entire genomes and within species.^13^ whole-genome bisulfite sequencing (WGBS) is particularly powerful, as it reveals genome-wide single nucleotide resolution of DNA methylation.^14^ WGBS has been used to sequence an increasing number of plant methylomes, ranging from model plants like A. thaliana^14^ to economically important crops like Z. mays.^15^ This has enabled a new field of comparative epigenomics, which places DNA methylation within an evolutionary context.^16^

In this study, *Medicago sativa* L. seeds were treated with plasma combined with plasma-activated water using a needle array-plate electric barrier discharge (DBD) device. DNA methylation analysis of seedlings was carried out to explore the effect of plasma treatment on *Medicago sativa* L. seeds at the molecular level, which provided experimental data supporting breeding via plasma.

## Results

### Survival rate

**Figure 1:**
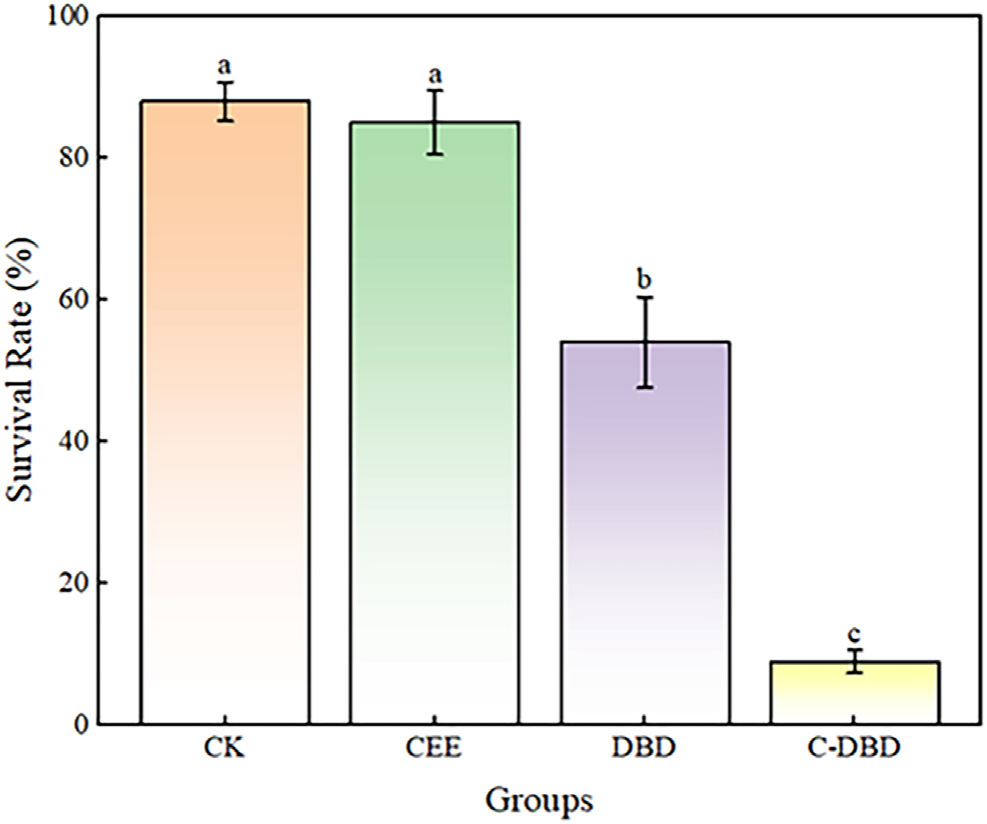
Survival rates (different letters indicate statistically significant differences between treatments (*p* < 0.05) Data are represented as mean ± SEM)

The survival rates of cultivated *Medicago sativa* L. cultivated under different treatment conditions are shown in Figure 1. The survival rates of the CK (control group), CEE, DBD, and C-DBD groups were 88%, 85%, 54%, and 9%, respectively. With the closure of the experimental environment and the addition of dielectric plates, the survival rate reached 9%. From the figure, it can be seen that there was no significant difference in the survival rate of seeds without a dielectric plate in a closed environment compared to the control group (*p* > 0.05), while there was a significant difference in the survival rate of seeds with and without a dielectric plate (*p* < 0.05).

### WGBS sequencing summary and QC

There was a significant difference in the traits between the CK group and the C-DBD group, which had the lowest survival rate of the *Medicago sativa* L. seedlings. Therefore, the C-DBD group (68 mg, Root length is 1.65 cm) and the CK group (45 mg, Root length is 1.06 cm) were selected for whole-genome methylation sequencing analysis, and gene ontology (GO) and path analyses were performed on genes related to the differentially methylated regions. The sample of *Medicago sativa* L. seedlings in the C-DBD group was named “mutant”, while the sample of *Medicago sativa* L. seedlings in the CK group was named “CK”. WGBS sequencing was performed on these two samples, with an average output of 124.825 Gb of filtered clean bases per sample. The offline data were filtered to obtain clean data. On average, each sample produced 124.825 Gb of filtered clean bases. The statistical results of the comparison are shown in Table 1. The average effective coverage rate of the entire genome was 82.38%, indicating high reliability and accuracy. The effective data coverage of the C site provides high-reliability information about the methylation status of individual cytosines. The sequence characteristics of C can be divided into three types, CG, CHG, and CHH, which affect the percentage of methylated cytosine. In addition, external treatments (cold plasma combined with plasma-activated water treatment) can also affect the percentage of methylated cytosine. There were significant differences in the types of regulatory elements (Table 2) and the distribution of methylation sites within each chromosome (Table S1).

**Table 1:** Reference genome alignment statistics

Sample	Clean reads	Clean rate (%)	Mapped reads	Mapping rate (%)	Uniquely mapping rate (%)	Bisulfites conversion rate (%)	Duplication rate (%)	Average depth (X)	Coverage (%)
CK	762149976	94.19	465650314	61.10	41.78	99.51	6.63	53.13	82.108
Mutant	902185902	93.23	578455246	64.12	43.89	99.43	7.81	64.72	82.643

**Table 2:** C locus coverage of samples within the range of various types of regulatory elements in the whole genome

Sample	Different Genome Regions	C (%)	CG (%)	CHG (%)	CHH (%)
CK	CDS	87.031	88.338	87.845	87.893
	Down2k	78.452	78.881	78.590	78.381
	Up2k	77.486	78.499	76.915	77.438
	mRNA	82.087	81.920	82.733	81.992
	repeat	78.483	79.760	78.457	78.324
	CpGlsland	78.486	79.179	78.715	78.100
Mutant	CDS	88.686	88.940	88.527	88.683
	Down2k	79.346	79.650	79.490	79.288
	Up2k	78.340	79.178	77.778	78.313
	mRNA	82.895	82.677	83.479	82.816
	repeat	79.512	80.673	79.425	79.379
	CpGlsland	78.851	79.537	79.084	78.465

CDS, coding sequence; Down2k, downstream 2 kb region of the genome; Up2k, upstream 2 kb region of the gene; mRNA, messenger ribonucleic acid; Repeat, genomic repeat sequence.

### Genome-wide patterns of DNA methylation in Medicago sativa L

Since *Medicago sativa* L. is multicellular, the methylation level of a C base ranges from 0% to 100%, which is equal to the number of sequences supporting mC covered by that C base divided by the total number of sequences effectively covered. CG methylation usually occurs in genes and repeat sequences and plays a very important role in gene expression regulation.^17^ Non-CG type sequences (CHG and CHH) are rare in genes, mainly in intergenic regions and regions rich in repeat sequences, and play a key role in silencing transposons.^18^ The average methylation level of the C-site sequence was calculated for different regions of the gene, including coding sequence (CDS), Down2k, Up2k, mRNA, and repeat regions (Table 3). For each region, the CG locus had the highest average methylation level among the three different sequence environments, while the CHH sequence environment exhibited the lowest methylation level. After cold plasma combined with plasma-activated water treatment, the DNA methylation level pattern of each genomic region remained unchanged, but the methylation rate changed. The methylation rates of the CHG and CHH types decreased slightly in the mRNA regions, the CHH type decreased slightly in the Down2k and Up2k regions, and the methylation rates of all types increased slightly in the other regions. In addition, the methylation levels of the samples were analyzed throughout the genome and within each chromosome (Table S2).

**Table 3:** Methylation levels of samples across genome-wide regulatory element types

Sample	Different Genome Regions	C (%)	CG (%)	CHG (%)	CHH (%)
CK	CDS	13.105	56.583	21.392	4.310
	Down2k	17.894	59.391	38.860	8.754
	Up2k	20.586	60.571	43.058	11.160
	mRNA	12.459	64.050	18.366	4.701
	repeat	31.592	90.151	77.458	15.061
	CpGlsland	34.625	72.534	52.322	10.105
Mutant	CDS	13.452	57.663	22.151	4.499
	Down2k	17.815	60.075	39.287	8.550
	Up2k	20.470	61.596	43.814	10.843
	mRNA	12.603	64.695	19.074	4.725
	repeat	31.134	90.168	76.277	14.695
	CpGlsland	35.556	74.967	53.526	10.565

**Figure 2:**
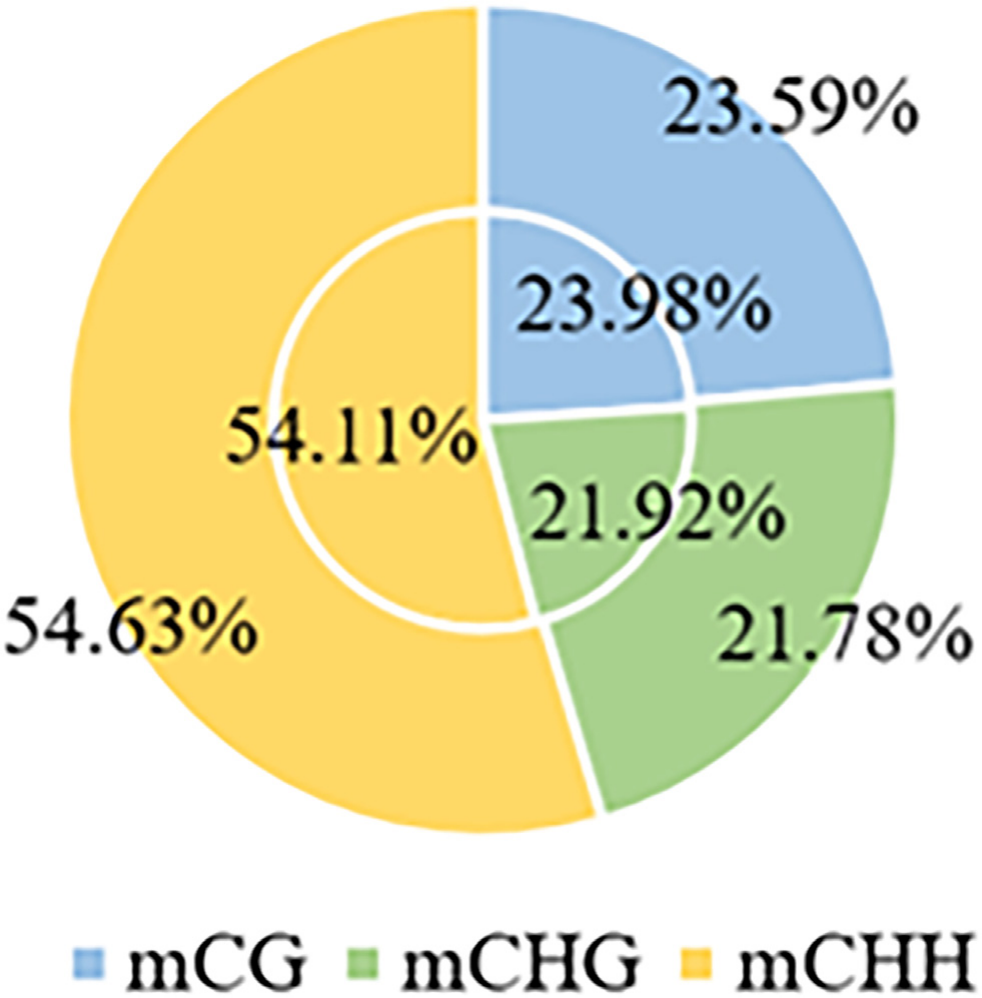
Relative proportion of cytosine methylation in different sequence environments (CK in the inner circle and mutant in the outer circle)

By analyzing the proportion of mCG, mCHG and mCHH sequences to the total mC loci, the distribution of mC loci in the three sequence environments can be obtained, which reflects the characteristics of genome-wide methylation patterns of specific species to some extent. The total proportion of the three base types (mCG, mCHG and mCHH) was 100%, and the methylated C identification method was carried out according to the method described by Lister.^18^ As shown in Figure 2, methylated cytosine occurred most frequently at the mCHH site (54.11%) and less frequently at the mCG and mCHG sites (23.98% and 21.92%, respectively). mCHH methylation slightly increased and mCG and mCHG methylation decreased after treatment with cold plasma and plasma-activated water, indicating that the level of DNA methylation remained relatively stable within a certain range.

**Figure 3:**
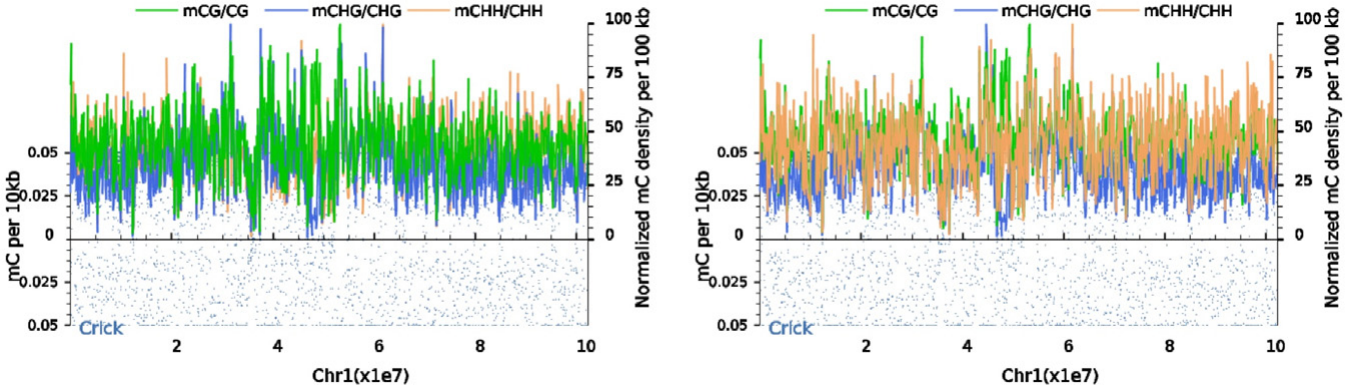
Distribution of methylation C base density at the chromosome 1 level (CK on left, mutant on right, Chr1 chromosome on horizontal axis, from left to right). The vertical axis on the left shows the mC density calculated from the 10 kb window, the distribution of mC density on chromosomes is shown as blue dots, the vertical axis on the right shows the normalized mC ratio, and the smooth curve shows the density distribution of different types of methylated C bases (CG, CHG, and CHH). The black part on the horizontal axis indicates the centromere.

As shown in Figures 3, S1, and S2, a global view of DNA methylation levels shows that the density of DNA methylation levels varies greatly from chromosome to chromosome. Under the combined treatment of cold plasma and plasma-activated water, the methylation levels of mCHH on chromosomes 1, 6, and 7 increased, the methylation levels of mCG and mCHG on chromosome 2 increased, the methylation levels of mCHH on chromosomes 4 and 5 decreased, and the methylation levels of other mC sequences and other chromosomes did not significantly change. In addition, DNA methylation levels are generally greater in the subtelomere regions of chromosomes, a phenomenon that is important for telomere length and recombination, as well as gene expression and protein-DNA interactions.^17^

**Figure 4:**
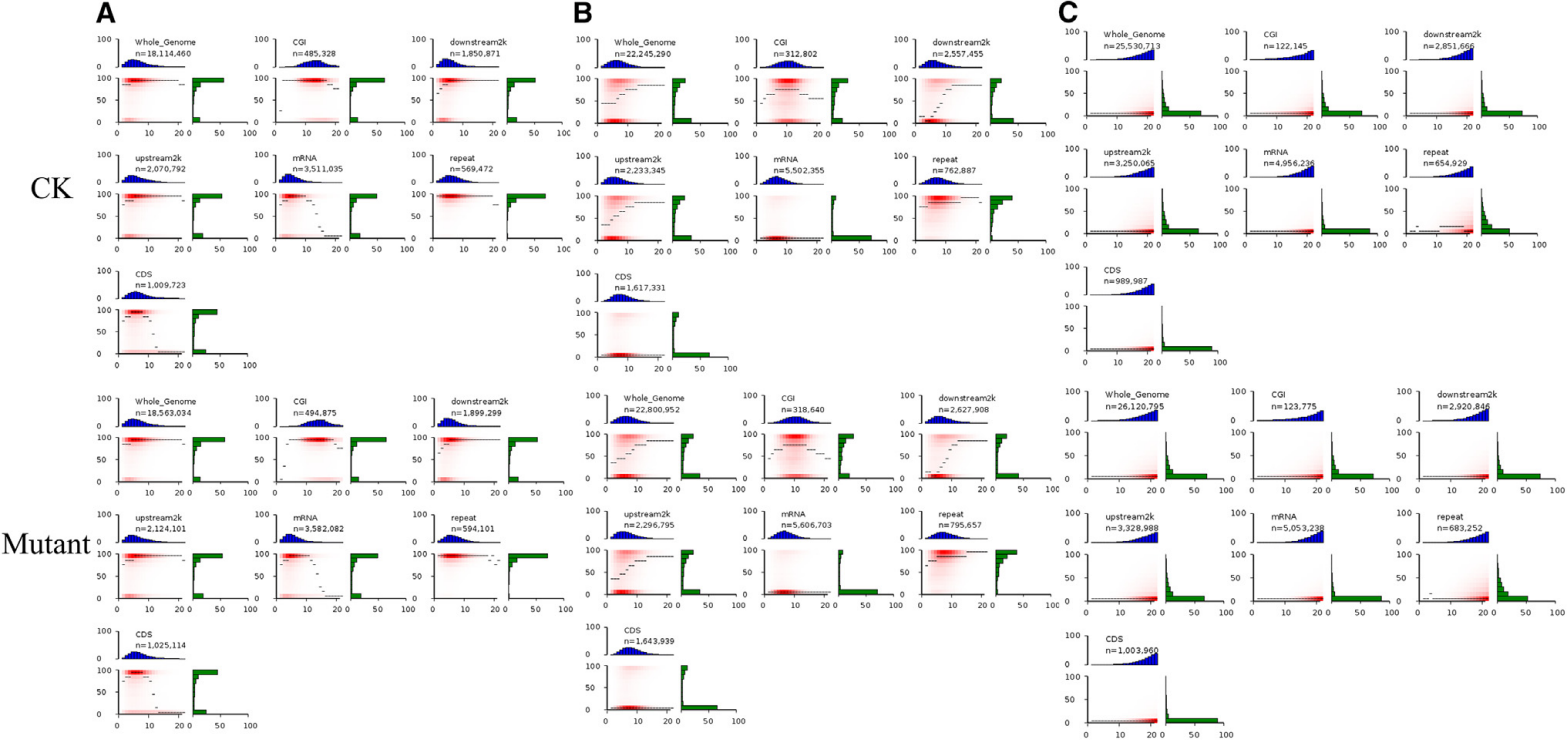
Distribution of methylation and CpG density in different regions of the genome In the lower-left heatmap, CpG density (X axis) is defined as the number of CpGs in a 200 bp window, and the vertical axis represents the average methylation level of the CpGs. The thin black lines in the graph represent the median methylation levels for such windows at a given CpG density. The red areas, from light to dark, represent CpG numbers at specific methylation levels and CpG densities. The blue bar graph at the top shows the distribution of CpG density mapped onto the horizontal axis, and the green bar graph at the right shows the distribution of methylation levels mapped onto the vertical axis. (A), (B), and (C) CG, CHG, and CHH sequences, respectively.

Different regions of the genome have different methylation patterns and perform different biological functions.^19^ Methylation levels in characteristic regions of the genome are represented in the form of heatmaps^20^. As shown in the Figure 4, mCG occurred most frequently in each genomic region and under two treatments (control group and cold plasma combined with plasma-activated water treatment), indicating that mCG loci were the most important type among the three sequence backgrounds.

**Figure 5:**
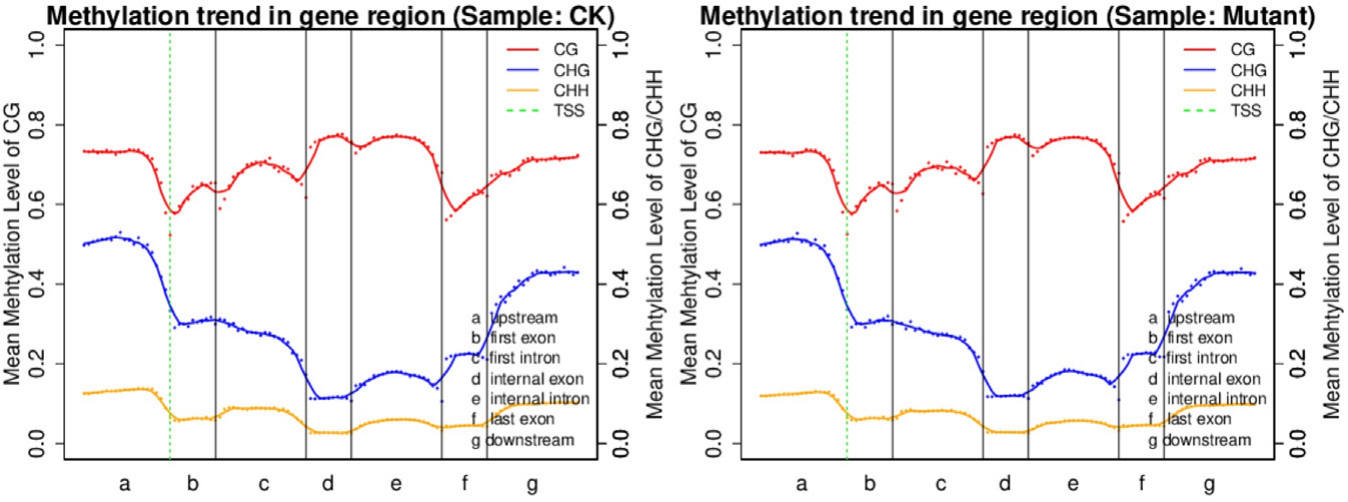
Distribution of methylation levels in different functional element regions of the genome The X axis shows the 7 different transcription element regions into which the whole gene is divided. The length of each transcription element region is divided into an equal number of bin regions (referring to the bin region containing a certain number of bases). Each point is the average methylation level of a bin region. The color curves represent the five-point average of the methylation level of each bin region. The vertical axis represents the methylation level (values from 0 to 1). The green dotted line between a and b is the TSS (transcription start site) position.

The distribution of DNA methylation levels in different functional regions is helpful for understanding the role of DNA methylation in different regions at the genome-wide level.^21^ As shown in Figure 5, the methylation level of mCG was greater than that of mCHG for different functional elements in both samples, and the methylation level of mCHH was the lowest. Except for the first intron, the methylation level of mCG in the mutant group was greater than that in the CK for different functional elements. In addition, the methylation sites of mCHG and mCHH had the same regularity, with high methylation at upstream and upstream TSSs, first introns, internal introns, and downstream elements and low methylation at downstream exons, internal exons and last exons of the TSS.

### Analysis of the preference for cytosine DNA methylation sequences

**Figure 6:**
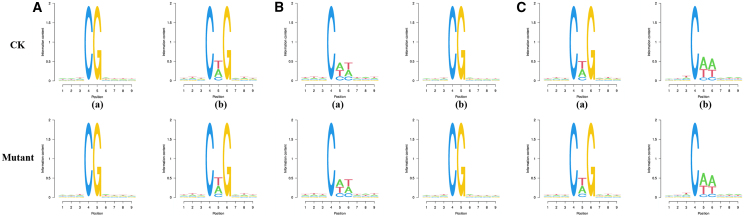
Sequence preference for cytosine methylation in Medicago sativa L. Weblogo analysis of bases around mC sites in different sequence backgrounds The X axis shows the position of the methylated cytosine at position 4, and the Y axis shows the entropy of the base. 0 is the minimum value, indicating that the proportion of four bases is uniform, all of which are 25%. 2 is the maximum value, indicating that the distribution of four bases is the most uneven; that is, only one specific base appears, such as C at the fourth position and G at the fifth position. (A), (B), and (C) CG, CHG and CHH sequences, respectively. Furthermore, a and b represent all C sites and mC sites, respectively.

In some eukaryotes, the sequence characteristics of bases near methylation sites have guiding significance in reflecting the sequence bias of methylation occurrence.^19^ In the genome of *Medicago sativa* L., all methylated mC sites have different sequence preferences around methylation sites in both the mCG and non-CG backgrounds (Figure 6). In symmetric CG environments, mC often appears in CTG sequences, methylation of mC sites in the mCHG background most frequently occurs in TCGA sequences, and methylation within the mCHH range most commonly occurs in CAA sequences. Therefore, it is inferred that the majority of methylation at the mC site occurs in the CNA (N represents A, T, G, C) sequence.

### Detection of differentially methylated regions (DMRs)

**Figure 7:**
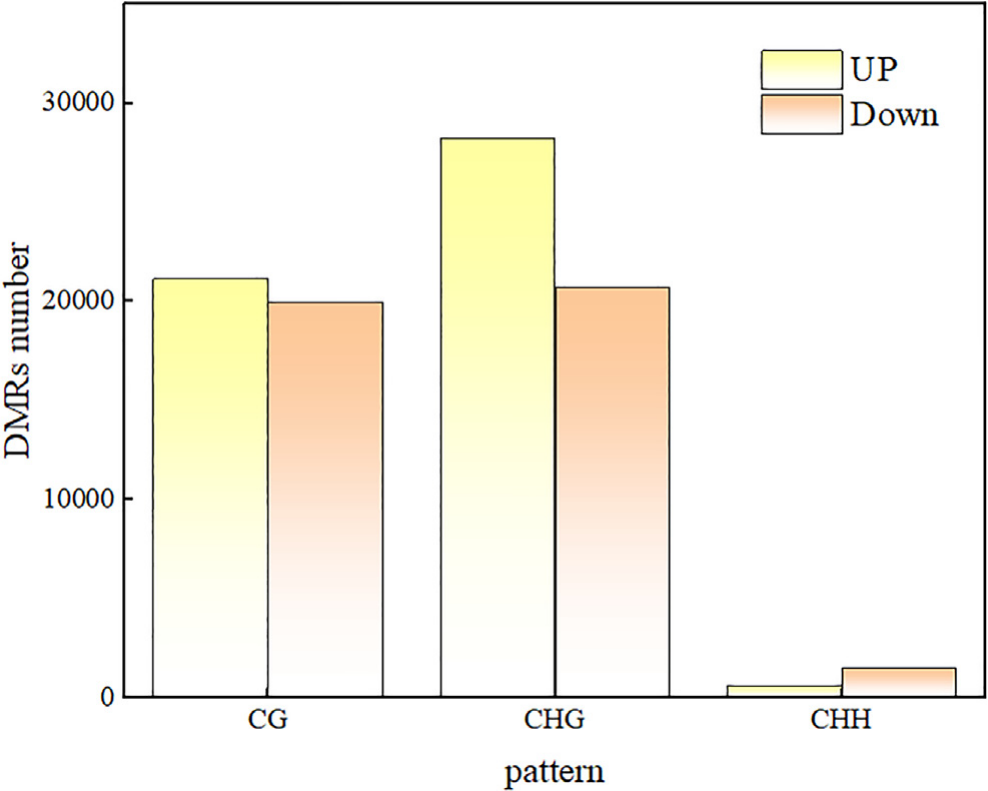
DMR statistics under different modes

DMRs refer to certain DNA segments that exhibit different methylation patterns in the genome in different samples. To reveal the potential role of methylation in response to cold plasma combined with plasma-activated water treatment, we compared treated groups with controls, identified DMRs containing up to 5 CG (CHG or CHH) sites using a sliding window approach, and evaluated the difference in methylation levels between the two samples.^20^ As shown in Table 4, compared with those in the control group, 41067, 48919, and 2046 DMRs were identified in the three sequence patterns of *Medicago sativa* L. treated with cold plasma combined with plasma-activated water. Analysis of DMR length revealed differences in the DMR length of chromosomes (chr) 8 in *Medicago sativa* L. When evaluating the mCG and mCHG modes, the longest DMR was found on chr5, while for the mCHH mode, the longest DMR was found on chr6. As shown in Figure 7, the methylation levels of DMRs increased and decreased, with 21132, 28232, and 584 DMRs increasing in the CG, CHG, and CHH modes, respectively, and with 19935, 20697, and 1462 DMRs decreasing in methylation levels. These results may be related to the genome, the number of transposons, and the presence of other elements on each chromosome. In addition, we infer that plants can affect gene expression to cope with external stress by altering certain genes or methylation patterns associated with stress.

**Table 4:** Statistical results of CK vs. mutant DMRs

Chr	mCG pattern		mCHG pattern		mCHH pattern	
	DMRs number	DMRs length	DMRs number	DMRs length	DMRs number	DMRs length
Chr1	5244	2125677	5557	2127375	268	55283
Chr2	4404	1767289	4704	1772707	206	39781
Chr3	5170	2067901	6124	2331335	247	49889
Chr4	4794	1938538	5265	1951827	242	46379
Chr5	5449	2234255	6180	2379731	273	54635
Chr6	5261	2092333	8467	3192234	294	61045
Chr7	4996	2010863	6088	2294098	208	41524
Chr8	4694	1863585	5379	1960749	257	51321
Fakechr1	1055	428792	1155	423310	51	10152
Total	41067	1659233	48919	1843366	2046	410009

### DMR-related gene enrichment analysis

GO and KEGG enrichment analyses of DMR-related genes obtained in the previous stage were carried out to explore the biological processes and pathway regulatory pathways related to the changes in *Medicago sativa* L. characters after cold plasma combined with plasma-activated water treatment and then to explore the regulatory role of differentially methylated genes in cold plasma combined with plasma-activated water treated.

**Figure 8:**
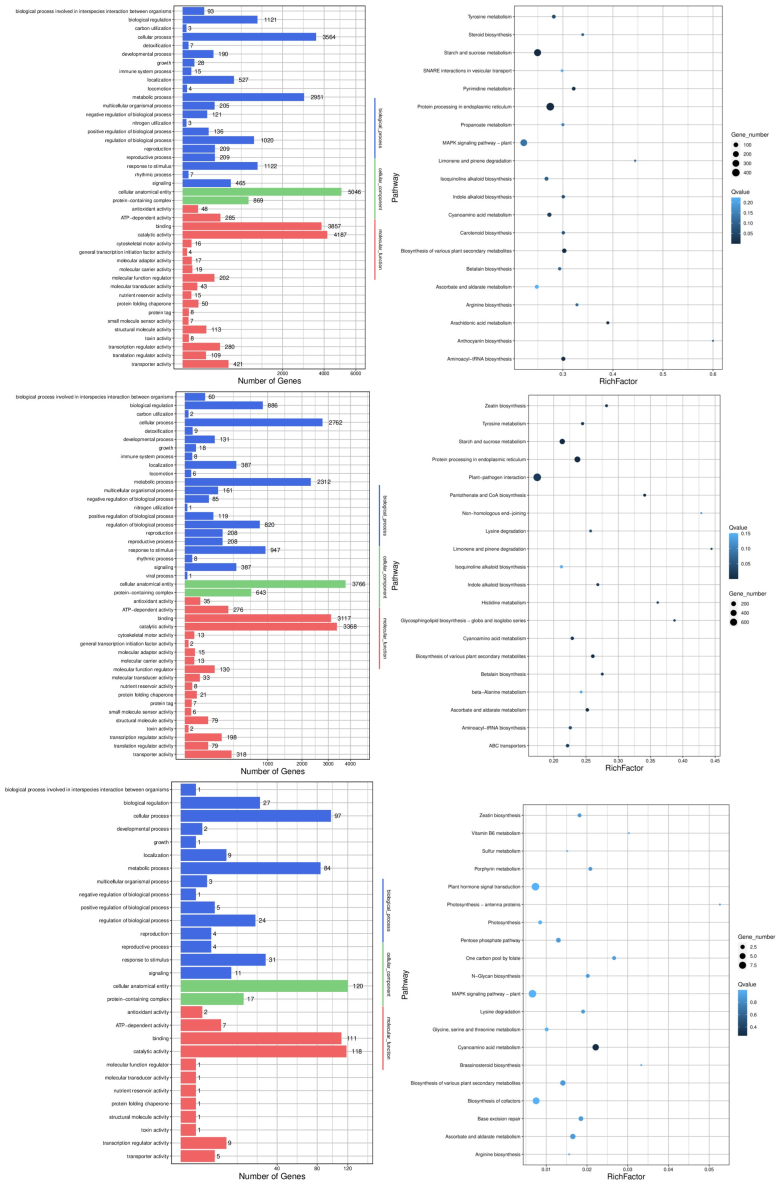
KEGG pathway enrichment and GO analysis of the DMG The horizontal axis of the graph on the left represents the number of DMGs, while the vertical axis represents various GO terms. All GO terms are categorized into three categories: blue represents biological processes, green represents cellular components, and red represents molecular functions. The rich factor in the right Figure represents the proportion of DMG to the total number of genes in the pathway, and the higher the RichFactor is, the more enriched the gene is in that pathway. Q is the corrected *p* value, which is 0–1. The smaller the Q value is, the greater the enrichment. Only the first 20 enriched pathways are shown in this Figure. The methylation patterns from top to bottom are CG, CHG, and CHH.

DMRs were annotated into gene body regions and enriched in three GO categories. The secondary annotations are shown in Figure 8, which are annotated into cellular components, molecular functions, and biological processes. The promoter region of genes affects transcriptional regulation, and differentially methylated promoters (DMPs) may affect transcription. To explore the mutagenic effect of cold plasma combined with plasma-activated water treatment on *Medicago sativa* L., we annotated DMRs in the promoter region and conducted GO enrichment analysis (Figure S3). The results showed consistent patterns of annotation in two regions and three methylation patterns. DMGs (differentially methylated genes) are highly enriched in cellular and metabolic processes (cellular components), cellular anatomical entities (molecular functional items), and connectivity and catalytic activity (molecular functional items).

KEGG enrichment analysis (Figure 8; Figure S3) revealed that CK vs. mutant was significantly enriched in 22, 23 and 2 pathways annotated to gene bodies and 15, 10, and 6 pathways annotated to promoters in the three methylation patterns, respectively (*p* < 0.05). Pathways annotated to gene bodies and significantly enriched in all three methylation patterns included cyanoamino acid metabolism pathway ID: ko00460 and ascorbate and arabinose metabolism pathway ID: ko00053, involving 74 and 60 DEGs (CG pattern), 62 and 61 DEGs (CHG pattern), and 6 and 4 DEGs (CHH pattern), respectively. Protein processing in the endoplasmic reticulum (ID: ko04141) was the pathway annotated promoter and significantly enriched in all three methylation patterns, involving 300 DEGs (CG pattern), 239 DEGs (CHG pattern), and 20 DEGs (CHH pattern), which may be closely related to the response of *Medicago sativa* L. to cold plasma combined with plasma-activated water mutagenesis.

## Discussion

**Figure 9:**
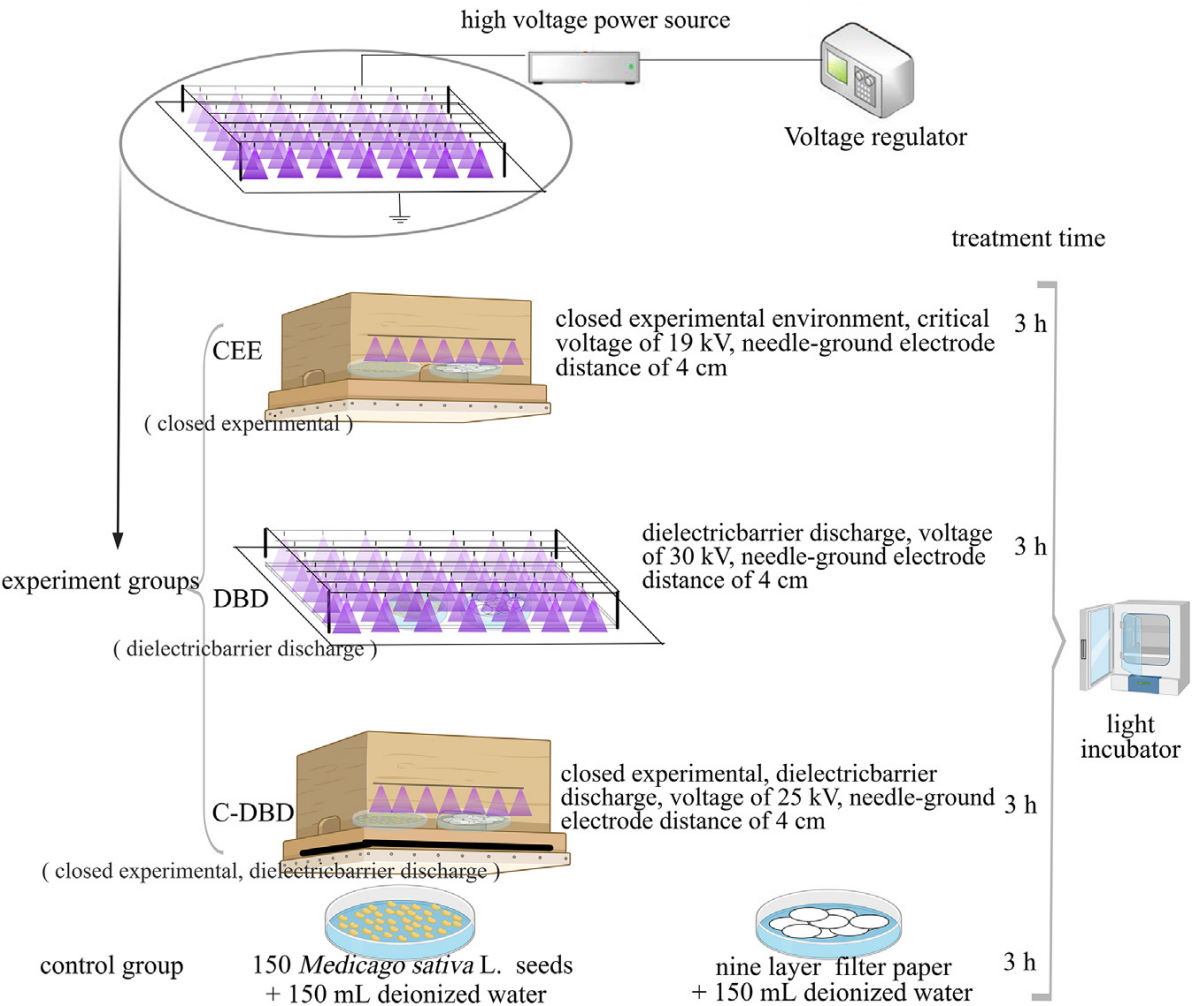
Medicago sativa L. treatment method

In this study, we established closed experimental environment and incorporated dielectric barrier plates (Figure 9), the survival rate of *Medicago sativa* L. seeds decreased to achieve successful mutation breeding. The survival rate of the experimental group was lower than that of the control group (CK> CED>DBD>C-DBD).

**Figure 10:**
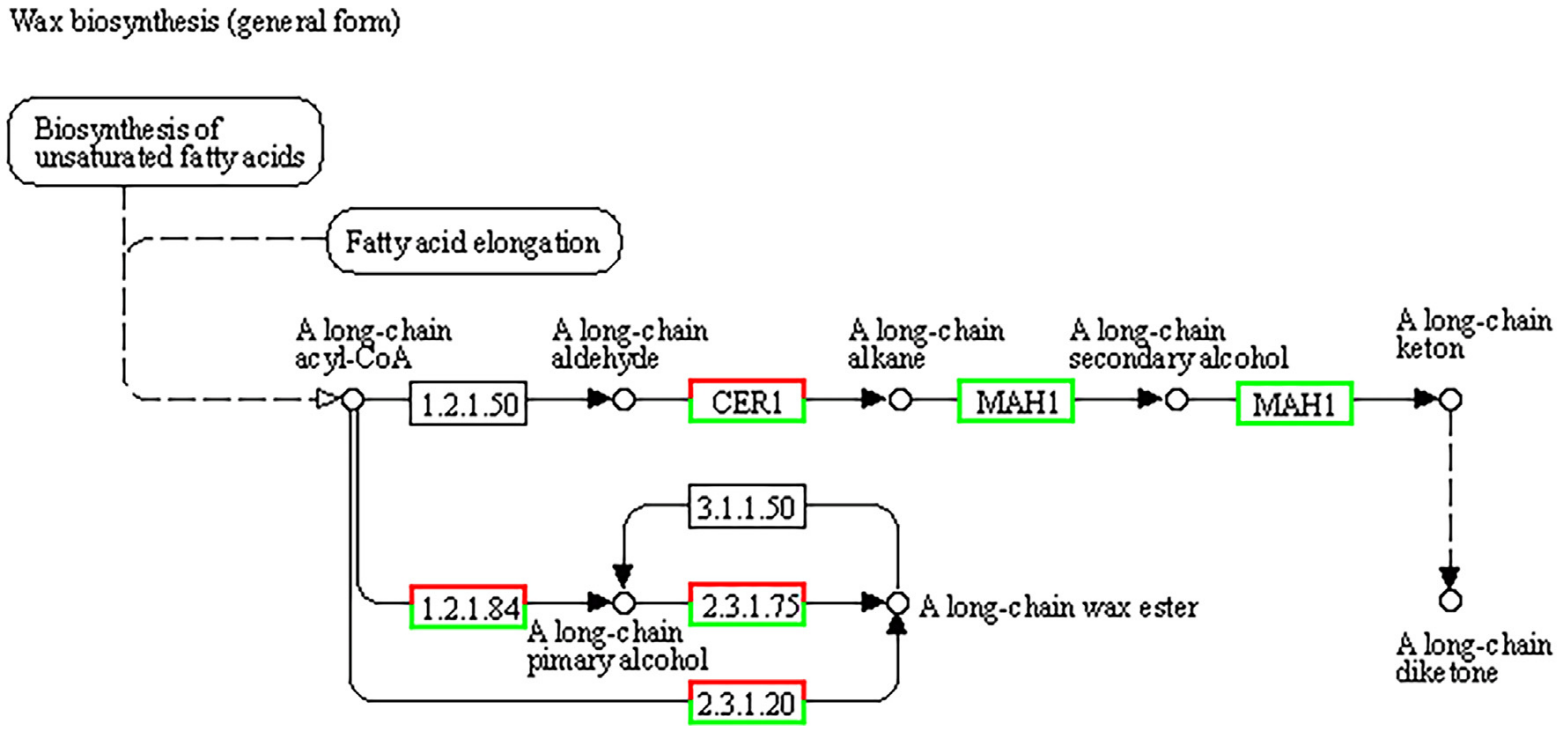
KEGG signaling pathway enrichment analysis of methylation-related genes in the wax biosynthesis pathway (red indicates upregulated gene expression, green indicates downregulated gene expression)

The fresh weight of the plants in the experimental group was significantly greater than that of the control group, potentially due to the downregulation of genes involved in the plant epidermal wax biosynthesis pathway. Given that the greatest number of genes are annotated to the gene body region in the CG mode, the wax biosynthesis pathway is effectively demonstrated in the context. Epidermal wax plays a crucial role in protecting plants from adverse external conditions, maintaining tissue and organ functions, and ensuring normal plant development.^21^ As shown in Figure 10, the expression of genes involved in the wax biosynthesis pathway was generally downregulated, resulting in a decrease in wax biosynthesis. The wax surface does not block water, facilitating the entry of water containing reactive oxygen and nitrogen species (RONS) into the plant body, which contributes to the increase in fresh weight.

WGBS has established itself as the “gold standard” for DNA methylation detection due to its high accuracy, reliability, and single-base resolution.^22^^,^^23^ In this study, C locus coverage increased for all types of regulatory elements and chromosomes across the whole genome following treatment with plasma combined with plasma-activated water. Currently, among the 34 plants analyzed for genomic DNA methylation, mCG exhibits the highest level of DNA methylation in the whole genome, *Arabidopsis* diaplays the lowest mCG level (30.5%), while *Beta vulgaris* has the highest 92.5%. CHG site methylation varies from 9.3% in *Eutrema salsugineum* to 81.2% in *Bate vulgaris*, and *Vitis vinifera* shows the lowest mCHH content (1.1%), with *Bate vulgaris* again having the 18.8%.^24^ Previous studies have indicated that mCG methylation levels are the highest, and *Medicago sativa* L. methylation levels conform to this pattern. Among various species, *Medicago sativa* L. demonstrates moderately high methylation levels. According to earlier research, genomic DNA methylation patterns change in response to abiotic stress.^11^^,^^25^ *Medicago sativa* L. treated with plasma and plasma-activated water exhibited methylation rates of approximately 21.738%, 76.355%, 48.875%, and 9.716% in C, CG, CHG, and CHH patterns, respectively. All these values differed from the control group, in accordance with the established trends. Our findings align with the previous methylome research, indicating that mCHH methylation is the highest among the three types, while mCG and mCHG methylation levels tend to be lower. Additionally, plasma and plasma-activated water treatments led to a slight decrease in the ratio of mCG and mCHG methylation, alongside an increase in mCHH methylation.

Sub-telomeric regions of chromosomes exhibited higher DNA methylation densities, which may be crucial for telomere length regulation and recombination.^26^ Moreover, the methylation status of mCG, mCHG, and mCHH differed based on the age, location, and physiology of individual organism.^27^ In the *Medicago sativa* L. genome, approximately 70–90% of mCG methylation sites were methylated, while mCHG methylation sites ranged from 30 to 60%, and mCHH sites were within 0–10%. This trend was also observed in the genomic DNA of other species. Similar trends were also observed in Populus trichocarpa^28^ (percentages of mCG, mCHG, and mCHH methylation were 41.9%, 20.9%, and 3.25%, respectively) and Betula platyphylla^29^(percentages of mCG, mCHG, and mCHH were 42.6%, 28.8%, and 5.2%, respectively). In Arabidopsis thaliana,^30^ the percentage of methylation of mCG, mCHG, and mCHH were 24.60%, 6.98%, and 1.70%, respectively. These findings suggest that methylation levels exhibit fluctuations in response to changes in the external environment.

DNA methylation status varies depending on the genomic region being examined, including CDS, Down2k, Up2k, mRNA, repeat, and CpG island regions. Generally, mCG methylation occurs within both genes and repetitive sequences, playing a crucial role in the regulation of gene expression. In this study, repeat sequences exhibited the highest levels of methylation among the genomic regions evaluated, consistent with prior research on mulberry trees.^26^ By methylating repeats, plants can modulate the expression levels of associated genes, thereby adapting to diverse environmental conditions. For instance, under stress conditions, plants may alter the methylation state of repeats to regulate the expression of stress resistance genes. Nonetheless, further in-depth studies are necessary to elucidate the specific mechanisms and biological functions associated with repeated methylation.

In certain eukaryotic organisms, cytosine methylation is associated with its nearest sequence context.^31^ Analysis reveals that most mC methylation occurs in the CNA context, with no significant difference observed between normally grown *Medicago sativa* L. and those exposed to abiotic stress. This indicates that the sequence preference for DNA methylation demonstrate a degree of conservation and stability across organisms. Even under external pressures, this preference remains unchanged, suggesting that methylation modifications are subject to stringent genetic and epigenetic regulation. This stability contributes to the maintenance of genetic information integrity and accuracy, ensuring that organisms can effectively sustain normal physiological functions and metabolic activities in response to stress. We also analyzed the proportions of mCG, mCHG, and mCHG sequences relative to total mC sites, observing a slight upregulation of mCHH methylation following treatment. A comparison of the DNA methylation density distributions of different chromosomes before and after treatment, revealed significant variability in methylation density across each chromosome.

DMRs are involved in genetic imprinting because they can be methylated in according to either the maternal or paternal chromosome. The methylated allele is often, but not always, the silenced allele. Differences between the methylation patterns in the parental chromosomes and the offspring’s chromosome are considered epigenetic lesions.^32^ The detection of distinct methylation regions revealed that the methylation levels of most DMRs increased across the three methylation patterns, with the CHG pattern exhibiting the highest number of DMRs. GO analysis indicated that these genes were primarily enriched in cellular processes, cellular anatomical entities and catalytic activities, with the largest number of genes annotated to the CG methylation pattern among the three patterns. Enrichment analysis identified that these genes were mainly involved in cyanoamino acid metabolism (pathway ID: ko00460), ascorbate and aldarate metabolism (pathway ID: ko00053), and protein processing in the endoplasmic reticulum (pathway ID: ko04141). Recent studies have shown that cyanide is a highly toxic substance due to its ability to inhibit cellular respiration. Plants can produce and utilize cyanide, which plays a significant role in growth, development, and stress responses.^33^ Ascorbic acid (AsA), also known as vitamin C, is a water-soluble antioxidant organic molecule ubiquitous in plants. Its primary function is to detoxify. Intracellular oxides and superoxide, contributing to the stress response.^34^ When AsA is highly enriched, it may indirectly influence methylation levels by regulating other metabolic pathways or signaling cascades associated with DNA methylation. For example, ascorbic acid may affect the folate cycle or one-carbon metabolism pathways, both of which are crucial for DNA methylation. The survival rate of *Medicago sativa* L. seeds in the C-DBD group was only 9%, which may be related to the excessive reactive oxygen species (ROS) produced by plasma-activated water entering the seeds. Excessive ROS disrupt the ROS scavenging system, which protects the balance of the enzyme system,^35^ leading to high AsA enrichment. Protein processing in the endoplasmic reticulum is a complex process that requires the coordination of multiple molecular mechanisms and machines. This process is crucial for the normal operation of life systems, and abnormalities in these systems may also lead to the occurrence of certain diseases. These findings indicate that changes in DNA methylation across the plant genome are closely related to plant responses to abiotic stress.^36^

In addition to these highly enriched metabolic pathways, several noteworthy enriched pathways include the biosynthesis of various plant secondary metabolites (Pathway ID: ko00999) and indole alkaloid biosynthesis (Pathway ID: ko00901), both of which are closely related to the synthesis of flavonoids and terpenoids in *Medicago sativa* L. It was concluded that gene expression regulated by DNA methylation is one of the mechanisms through which plasma combined with plasma-activated water treatment influences the biosynthesis of active components in *Medicago sativa* L. Recent studies increasingly demonstrate that the synthesis and accumulation of secondary metabolites serve as a crucial defense strategy for medicinal plants against environmental stress, resulting in the accumulation of these metabolites, which typically represent the main medicinal components of such plants.^37^ Additionally, starch and sucrose metabolism (Pathway ID: ko00500) was prominently featured. Sucrose metabolism not only plays a role in plant growth and development but also responds to various of abiotic stresses. Understanding the underlying response mechanisms is critical for enhancing plant tolerance to stresses such as drought, high temperature, and cold.^38^ This study lays the groundwork for further exploration of mutation breeding in *Medicago sativa* L. The specific mediating mechanisms will necessitate additional experimental data for clarification. This marks the first comprehensive presentation of DNA methylation changes occurring in *Medicago sativa* L. in response to plasma and its plasma-activated water treatment stress. The experimental data provided will contribute to future studies on the relationship between DNA methylation and gene expression in plants facing abiotic stresses such as plasma and its activated water.

### Limitations of the study

The limitation of this study is that there is no decipherment of the actual mechanisms involved, and there is not much data that can be realistically commented on or scientifically analyzed. The main reason for this problem is that methylation sequencing alone may not fully reveal the complex mechanisms by which organisms respond to stress. DNA methylation is often involved in biological stress responses by influencing gene expression, but the regulation of gene expression is a multi-layered and complex network involving multiple regulatory elements such as transcription factors, MicroRNA, and Long Noncoding RNA. Therefore, methylation sequencing results need to be combined with other omics data (e.g., transcriptome sequencing, proteome sequencing, etc.) for comprehensive analysis. However, this sequencing method maximizes the complete genome-wide methylation information, covering the entire genome-wide methylation region. At the same time, methylation sequencing technology has extremely high resolution to accurately analyze the methylation status of each C base. In the next step of the study, we will perform other sequencing methods, joint analysis, and decipher their mechanisms.

## Resource availability

### Lead contact

Further information and requests for resources and reagents should be directed to and will be fulfilled by the lead contact, Hao Chen (slchen1126@126.com).

### Materials availability

This study did not generate unique reagents and is not part of a clinical trial.

### Data and code availability

• The whole-genome bisulfite sequencing data generated in this study has been deposited in NCBI sequence read archive (SRA) under Bioproject PRJNA1103876. The data are open to https://www.ncbi.nlm.nih.gov/bioproject/PRJNA1103876.
• All original code has been deposited at NCBI sequence read archive and is publicly available as of the date of publication.
• Any additional information required to reanalyze the data reported in this paper is available from the lead contactupon request.

## Acknowledgments

The authors are grateful for the support provided by the 10.13039/501100001809National Natural Science Foundation of China (no. 12265021 and no.12365023), Research Program of Science and Technology at Universites of Inner Mongolia Autonomous region (no. NJYT23108), The basic scientific research business project of the universities directly of the Inner Mongolia Autonomous Region (no. JY20240068 and no. JY20220057), 10.13039/501100004763Natural Science Foundation of Inner Mongolia Autonomous Region (2024MS01001).

## Author contributions

Conceived and designed the experiments: F.X. and H.C.; performed the experiments: F.X., C.C., and J.L.; analyzed the data: F.X. and H.C.; contributed reagents/materials/analysis tools: Z.S. and C.D.; rote the paper: F.X. and H.C.

## Declaration of interests

The authors declare no conflict of interest.

## STAR★Methods

### Key resources table

**Table:** 

REAGENT or RESOURCE	SOURCE	IDENTIFIER
**Biological samples**		

*Medicago sativa* L.	Grassland Research Institute of the Chinese Academy of Agricultural Sciences	

**Deposited data**		

whole-genome bisulfite sequencing data	NCBI Sequence Read Archive PRJNA1103876	

**Software and algorithms**		

SPSS		
Origin		

### Experimental model and study participant details

The *Medicago sativa* L. variety “Zhongcao 3” used for experimentation was obtained from the Grassland Research Institute of the Chinese Academy of Agricultural Sciences. Mature, plump, and uniformly sized Medicago sativa L. seeds were carefully selected. The surface impurities were repeatedly rinsed with deionized water, and the moisture was removed. The seeds were then air-dried and set aside for later use.

### Method details

#### Treatment methods

In this study, we used a needle array dielectric barrier discharge device (Figure 9). The device combines a DBD structure with a traditional pin array plate corona discharge structure to obtain high-concentration atmospheric pressure and cold plasma, making it highly suitable for biomedical applications.^39^ The power supply for the experimental device was Alternating Current (AC), the frequency was 50 Hz, and the voltage was adjusted from 0 to 50 kV. The high-voltage electrode consisted of a needle array comprising 14 × 7 needles, each 2 cm in length, with a diameter of 1.56 (±0.02) mm and curvature radius of 0.75 mm. The horizontal and vertical spacing between the needles was 4 cm, and the grounding end featured a planar aluminum plate measuring 100 × 60 cm^2^ and 2 mm thick. Additionally, to prevent the evaporation of particles generated during discharge, which can reduce the concentration of ions reacting with water and affect the concentration of active particles in the activated water, a closed experimental environment was created.

We established three treatment groups. Group CEE: Closed experimental environment without a dielectric barrier and a critical voltage of 19 kV. Group DBD included an open experimental environment with a 6-mm plexiglass plate (100 × 60 cm^2^) and an electric field voltage of 30 kV. Group C-DBD: Closed experimental environment with a 6-mm plexiglass plate and an electric field voltage of 30 kV. The plasma treatment duration was 3 h. Following plasma treatment, 150 *Medicago sativa* L. seeds and nine filter papers were evenly distributed in three polyethylene culture dishes with a diameter of 9 cm. The dishes were wrapped three times with sealing film and placed in a light incubator set to a constant temperature of 24°C. The experimental environment temperature was 26°C ± 2°C, and the humidity was 35% ± 5% RH.

#### *Medicago* sativa L. Seed survival rate

After germination, 100 lux light was provided in the light incubator, subjected the seedings to continuous light for 14 h and darkness for 10 h. The survival rate was assessed 6 days after seed inoculation. *Medicago sativa* L. seedlings were considered to have survived when their total length exceeded 2 cm. Seed germination characteristics were calculated as follows:

$$S=\frac{n6}{N}\times 100\%$$

S: Survival rate; N: Total number of seeds; n6: Number of germinated plants on Day 6.

#### WGBS

First, the sample is extracted, and the specific method flow is as follows. In the first step, prepare a 2% hexadecyltrimethylammonium bromide (CTAB) lysis buffer, and add 2% β-mercaptoethanol. Incubate at 65°C. The samples consist of plant seedlings grown for 30 days in both the CK group and the C-DBD group. In the second step, transfer the sample to a 2.0 mL grinding tube, and grind with liquid nitrogen until a powder. Add 0.9 mL of a mixed solution containing 2% CTAB and 2% β-mercaptoethanol, then incubate at 65°C for 60 min while shaking, and allow to cool to room temperature. In the third step, centrifuge at 12,000 × g for 5 min. Transfer the supernatant to a new 2.0 mL centrifuge tube, add an equal volume of phenol/chloroform/isoamyl alcohol (25:24:1), and vortex to mix. Centrifuge at 12,000 × g for 10 min at room temperature. In the fourth step, transfer the supernatant to a new 2.0 mL centrifuge tube, add an equal volume of chloroform/isoamyl alcohol (24:1), and vortex to mix. Centrifuge at 12,000 × g for 10 min at room temperature.

The whole genome methylation library preparation workflow is as follows: First, a Bioruptor (Diagenode, Belgium) was used to break the genomic DNA into fragments with an average size of 250 bp. The ends of the DNA fragments were repaired, an A base was added at the 3′ end, and the DNA was connected to the methylation ligands. An EZDNA Methylation Gold Kit (ZYMO) was used for bisulfite treatment, 2% agarose gel electrophoresis was used for fragment selection, DNA fragments were retrieved with a QIQuick Gel Extraction Kit (Qiagen). Finally, qualified libraries underwent Polymerase Chain Reaction (PCR) amplification and subsequent machine sequencing.

After obtaining the DNA sample, it was initially tested for quality. Once the sample quality was deemed acceptable, it was untallied for BS library construction. Following this, the quality of the library was assessed. Only qualified libraries will be used for sequencing. Genome C base methylation information was calculated using unique alignment data, followed by information analysis to produce both standard and personalized analysis results. Figure S4 illustrates the analysis process.

### Quantification and statistical analysis

All experiments were repeated three times, and the experimental results were taken as the mean ± standard deviation of the three experimental data. One-way analysis of variance (ANOVA) was used to determine the differences in the survival rate of Medicago sativa L. seeds, and *p* < 0.05 was a significant difference.
