# Supplementary material for: Genome-wide DNA methylation analysis of Medicago sativa L. treated with plasma and plasma-activated water
Source: iScience. 2025 Jan 25;28(2):111901. doi: 10.1016/j.isci.2025.111901 (PMC11883386; doi:10.1016/j.isci.2025.111901)
Supplement: Document S1. Figures S1–S4, Tables S1, and S2 [file mmc1.pdf]

iScience, Volume *28*

Supplemental information

Genome-wide DNA methylation analysis of *Medicago sativa* L. treated with plasma and plasma-activated water

Fei Xu, Hao Chen, Chan Chen, Jiaqi Liu, Zhiqing Song, and Changjiang Ding

Figure S1. Methylation C base density distribution (CK on the left and mutant on the right)

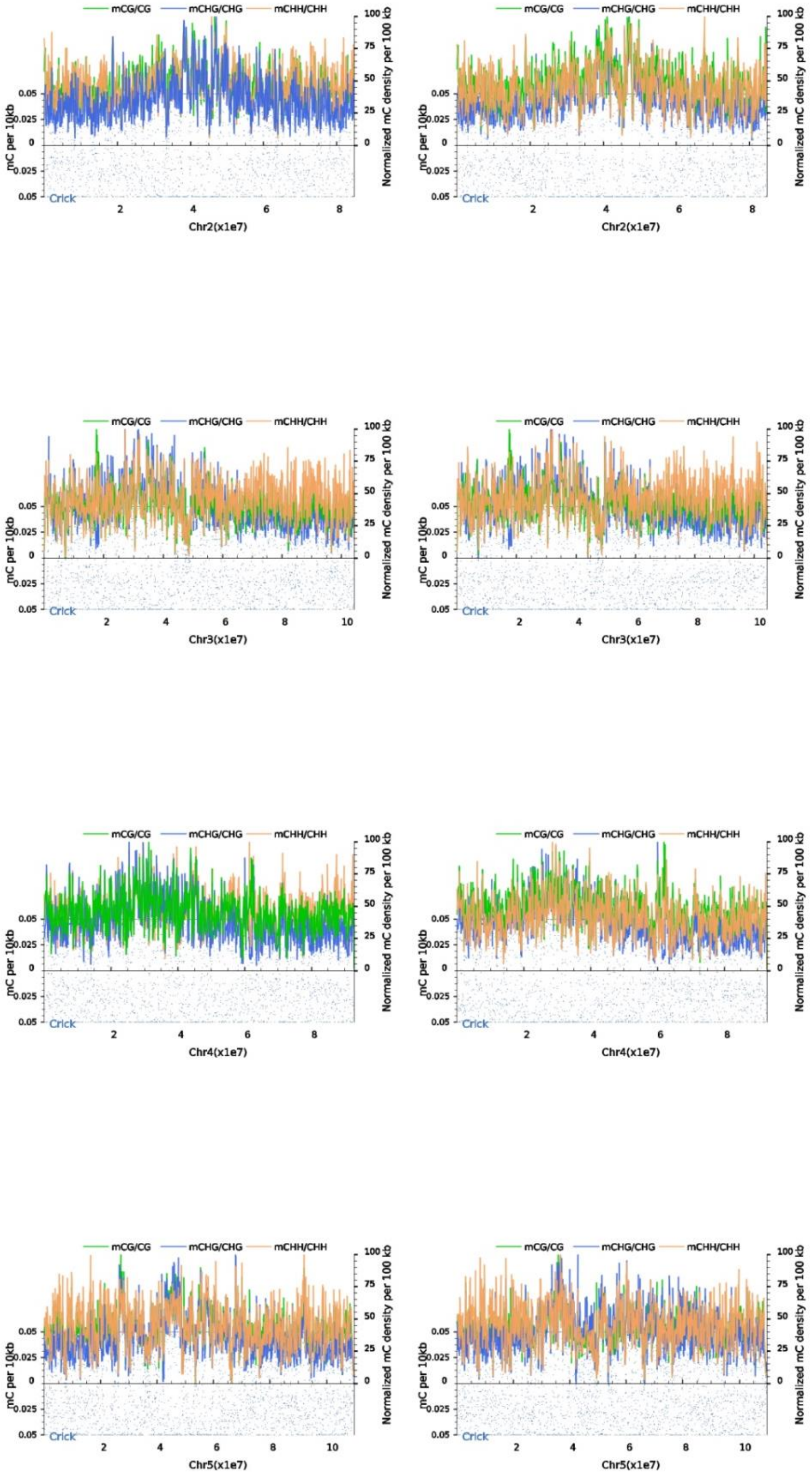

Figure S2. Methylation C base density distribution (CK on the left and mutant on the

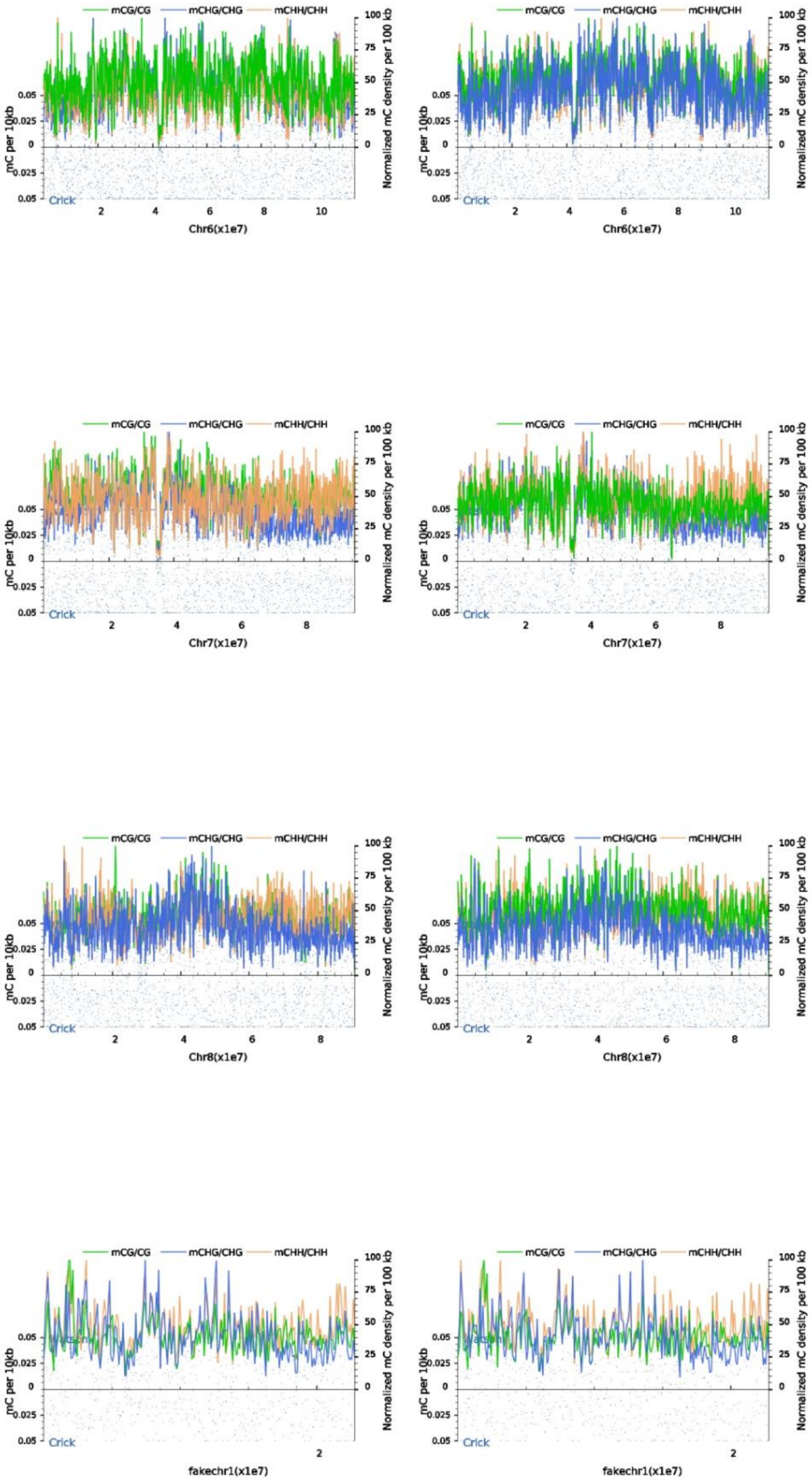

right)

Figure S3. KEGG Pathway Enrichment and GO Analysis of Related Promoters

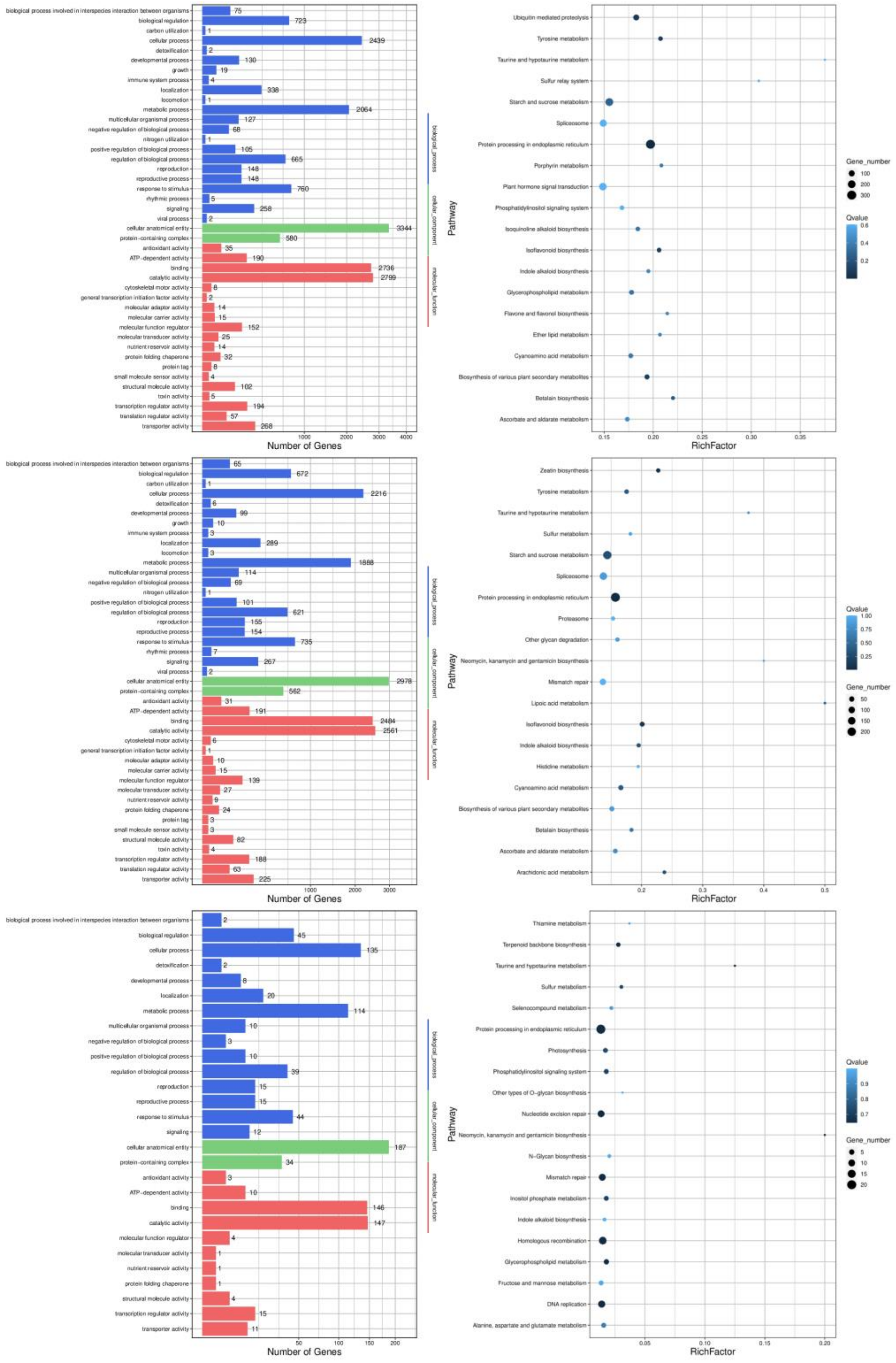

Figure S4. analysis process

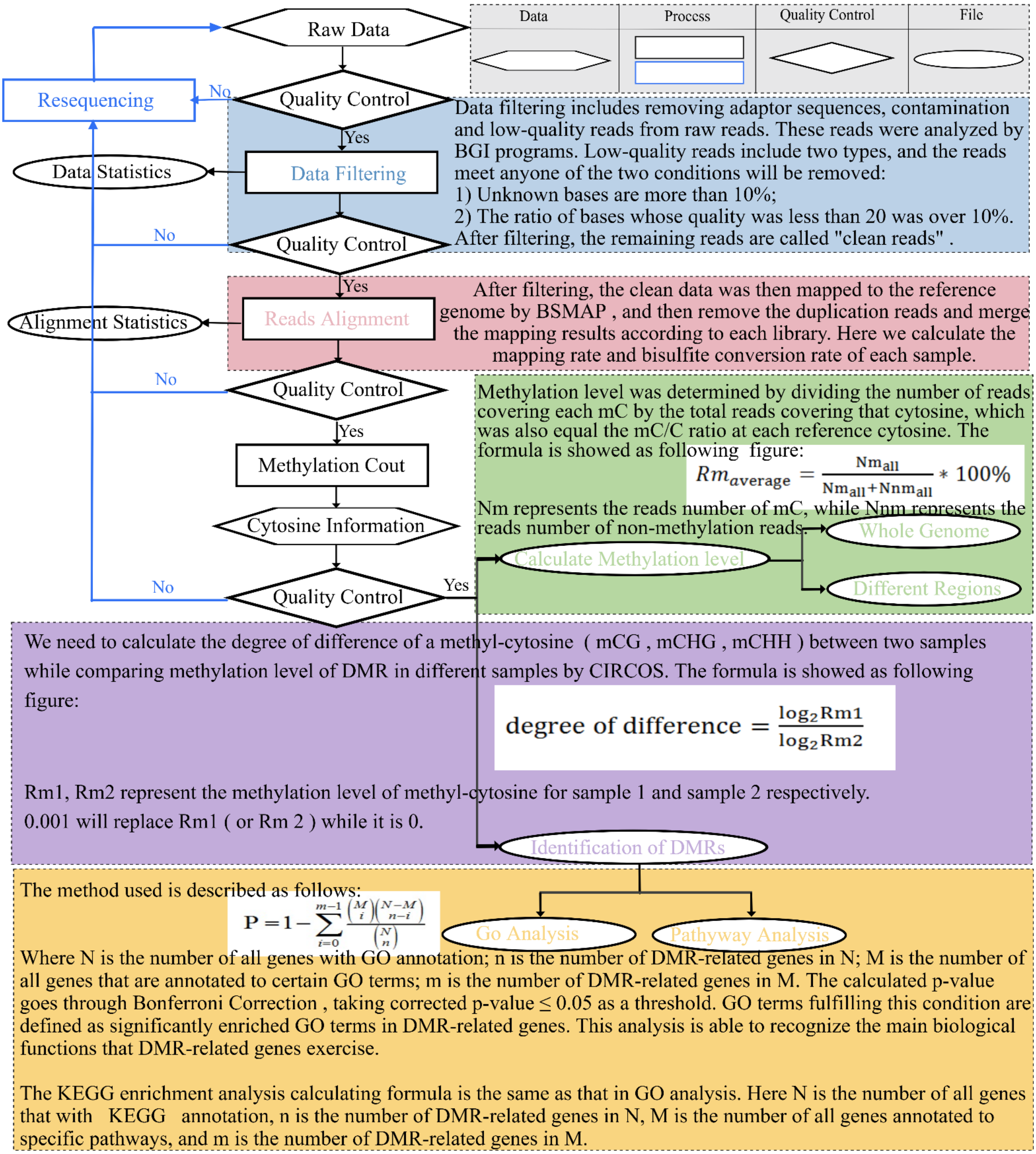

Table S1. C locus coverage of samples on each chromosome

Sample	Chromosome	C (%)	CG (%)	CHG (%)	CHH (%)
CK	Whole Genome	76.949	77.229	77.621	76.812
	Chr1	75.676	75.559	76.508	75.565
	Chr2	77.989	78.102	78.529	77.894
	Chr3	77.211	77.517	77.991	77.052
	Chr4	79.404	79.549	79.968	79.299
	Chr5	76.144	76.745	77.393	75.88
	Chr6	74.797	75.361	75.455	74.623
	Chr7	78.704	78.907	79.008	78.632
	Chr8	78.015	78.412	78.421	77.904
	Fakechr1	70.815	71.164	71.134	70.724
Mutant	Whole Genome	77.755	77.886	78.351	77.648
	Chr1	77.225	76.945	77.967	77.148
	Chr2	79.646	79.678	80.132	79.569
	Chr3	78.421	78.654	79.074	78.291
	Chr4	79.443	79.446	79.977	79.361
	Chr5	74.46	74.62	75.534	74.279
	Chr6	76.979	77.412	77.515	76.84
	Chr7	78.873	79.017	79.17	78.808
	Chr8	79.341	79.669	79.712	79.244
	Fakechr1	72.046	72.227	72.443	71.965

Table S2. Table Methylation levels of samples in genome-wide and chromosomal ranges

Sample	Chromosome	C (%)	CG (%)	CHG (%)	CHH (%)
CK	Whole Genome	21.900	76.047	48.817	9.909
	Chr1	22.336	78.898	50.31	10.046
	Chr2	22.431	77.275	49.783	10.723
	Chr3	19.251	64.382	41.841	8.701
	Chr4	21.69	77.373	47.581	10.137
	Chr5	22.612	78.486	51.721	10.404
	Chr6	25.021	84.772	59.303	10.268
	Chr7	22.034	77.737	49.272	10.118
	Chr8	21.264	74.22	44.952	10.389
	Fakechr1	19.184	72.255	35.375	6.886
Mutant	Whole Genome	21.738	·76.355	48.875	9.716
	Chr1	22.106	78.833	50.353	9.818
	Chr2	22.118	77.419	49.747	10.364
	Chr3	19.22	64.904	42.096	8.632
	Chr4	21.694	78.143	48.782	9.862
	Chr5	21.982	77.626	49.666	10.124
	Chr6	25.005	84.602	59.376	10.177
	Chr7	21.822	78.203	49.138	9.858
	Chr8	20.993	74.578	45.076	10.078
	Fakechr1	19.519	74.356	36.706	7.168
